# Low wall shear stress is associated with the rupture of intracranial aneurysm with known rupture point: case report and literature review

**DOI:** 10.1186/s12883-016-0759-0

**Published:** 2016-11-18

**Authors:** Yisen Zhang, Linkai Jing, Ying Zhang, Jian Liu, Xinjian Yang

**Affiliations:** Department of Interventional Neuroradiology, Beijing Neurosurgical Institute and Beijing Tian Tan Hospital, Capital Medical University, Beijing, 100050 China

**Keywords:** Rupture point, Hemodynamics, Intracranial aneurysms, Case report

## Abstract

**Background:**

Few previous hemodynamic studies demonstrated the detailed features of rupture point of intracranial aneurysms. The hemodynamic simulation for the case that ruptured during angiography was even rare. In the present study, we studied the hemodynamic characteristics of a posterior communicating artery segment aneurysm that ruptured during angiography and detailed the hemodynamic features at the rupture point.

**Case presentation:**

One 64-years-patient was 60–69 years old and suffered a subarachnoid hemorrhage within 24 h. Standard digital subtraction angiography and three-dimensional (3D) rotational angiography were performed and an 8 mm left posterior communicating artery segment aneurysm was found. The patient had a seizure immediately following 3D angiography for about 40 s and the immediate follow-up angiography showed contrast extravasation from the tip of identified aneurysms. The consequent vital sign of the patient became unstable. Urgent embolization under general anesthesia was planned, but the relatives refused interventional operation considering the high risk of procedure and poor condition of the patient. The computational fluid dynamic (CFD) method was used to evaluate the hemodynamic characteristics at rupture point, and the results showed that the rupture point was associated with markedly low wall shear stress and high oscillatory shear index without flow impingement.

**Conclusions:**

We present a rare case of which the rupture site was identified during angiography. The hemodynamic simulations revealed that the rupture point was associated with markedly low WSS and high OSI without flow impingement. The result may be unique to this particular aneurysm; however, our findings provide insight into the hemodynamics of rupture point.

**Electronic supplementary material:**

The online version of this article (doi:10.1186/s12883-016-0759-0) contains supplementary material, which is available to authorized users.

## Background

Few previous hemodynamic studies demonstrated the detailed features of rupture point of intracranial aneurysms and only 5 studies with 29 cases have been published previously [1–5]. The hemodynamic simulation for the case that ruptured during angiography was even rare [3, 4]. In the present study, we studied the hemodynamic characteristics of a posterior communicating artery segment aneurysm that ruptured during angiography and detailed the hemodynamic features at the rupture point.

## Case presentation

**Fig. 1:**
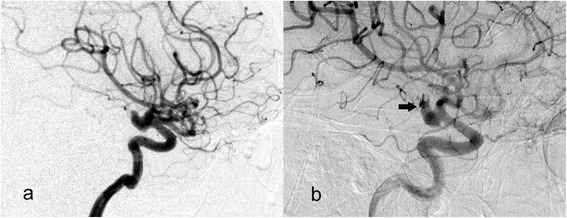
The angiograms performed before rupture (**a**) and immediately after rupture (**b**). The rupture point was identified at the tip of the aneurysm dome (*black arrow*)


A 64-year old man was admitted to our hospital due to a spontaneous sudden and severe headache with nausea and vomiting. The patient was healthy, with no prior history of hypertension or diabetes. A physical examination revealed that the patient was conscious and able to answer questions accurately, and that her limbs were flexible with normal muscle strength grade V, a positive Kernig’s sign, and a Hunt-Hess grade of II. Standard digital subtraction angiography and three-dimensional (3D) rotational angiography were performed and an 8 mm left posterior communicating artery segment aneurysm was found. The patient had a seizure immediately following 3D angiography for about 40 s and the immediate follow-up angiography showed contrast extravasation from the tip of identified aneurysms (Fig. 1). The rate of contrast emission was 3 mL/s with a total volume of 18 mL for the 3D rotational angiography, and 3 mL/s with a total volume of 5 mL for standard digital subtraction angiography; the pressure was 200 psi. An additional movie file shows this in more detail [see Additional file 1]. The consequent vital sign of the patient became unstable. Urgent embolization under general anesthesia was planned, but the relatives refused interventional operation considering the high risk of procedure and poor condition of the patient.

Additional file 1 Video presentation of contrast extravasation from the tip of identified aneurysms. (MOV 8989 kb)


The rupture point was determined by inspection of extravascular blood in 2D angiography and match on the 3D aneurysm geometry. The aneurysm volume geometry for this patient was obtained by three-dimensional angiographic data. The aneurysm model was imported into the ICEM CFD (ANSYS Inc., Canonsburg, Pa, USA) to create 2.5 million finite volume tetrahedral element grids with 3 prism layers elements for computational fluid dynamic (CFD) simulations. The software, ANSYS CFX 14.0 (ANSYS Inc.) was then used to solve the flow governing Navier-Strokes equations with the assumption of laminar, incompressible, and Newtonian blood flow. The density and dynamic viscosity of the blood were specified as 1060 kg/m^3^ and 0.004 N · s/m^2^, respectively. The blood vessel wall was assumed to be rigid with no-slip boundary conditions. The pulsatile velocity profile obtained by transcranial Doppler from a normal subject was applied for the inflow boundary conditions. Three cardiac cycle simulations were performed for numerical stability and the last cardiac cycle was collected as output.

The hemodynamic characteristics at rupture point and the whole aneurysm dome were calculated for comparison. In each area, peak systolic, end diastolic and time-averaged wall shear stress (WSS) was measured. Oscillatory Shear Index (OSI) was also calculated to investigate the changes in the WSS vector within the cardiac cycle. The intra-aneurysmal flow structure was also evaluated to determine the flow impingement and the contour of velocity was depicted on a cutting plane.

**Fig. 2:**
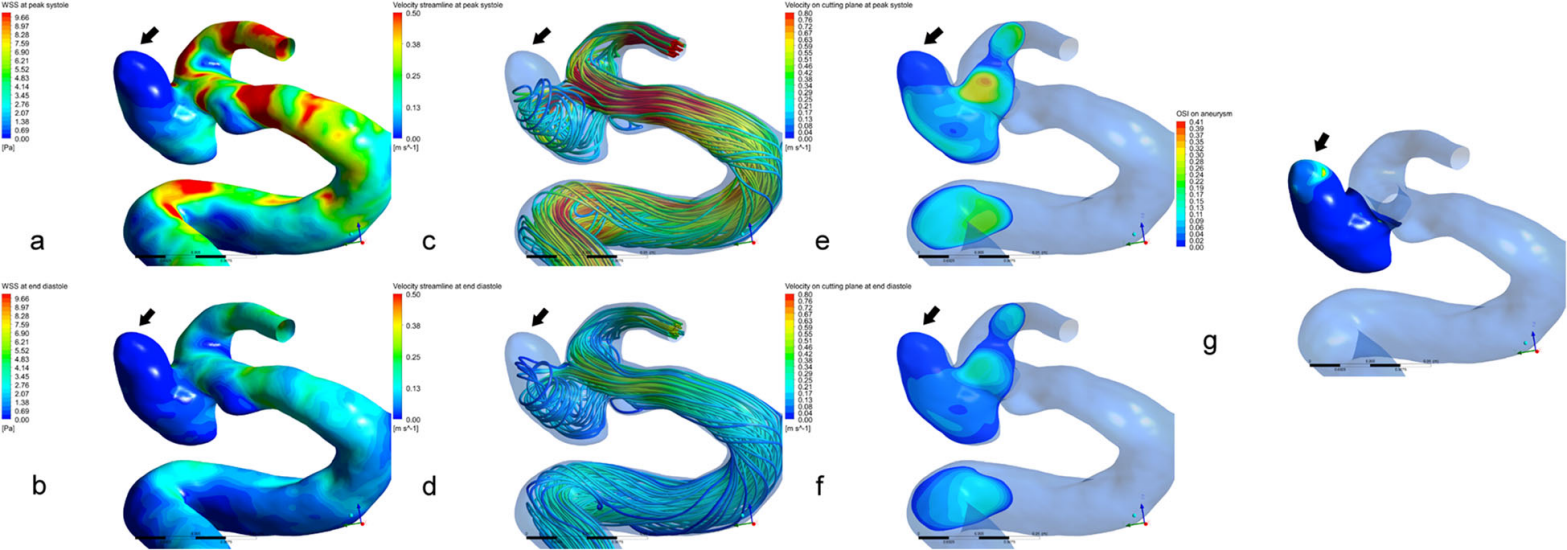
Hemodynamic characteristics of the aneurysm dome and rupture point just before the rupture. *Black arrows* indicated the rupture point. The hemodynamics of the case at peak systole was demonstrated at the first row and that at end diastole were at the second row. Wall shear stress (WSS, **a** and **b**), intra-aneurysmal streamline (**c** and **d**), velocity at a cutting plane (**e** and **f**) and the oscillatory shear index (OSI, **g**) were demonstrated respectively. Low WSS and high OSI were found at the rupture point without flow impingement

Demonstrated in Fig. 1, the rupture point was identified at the distal tip of aneurysm dome. The area of rupture point showed a low WSS distribution both at peak systole and end diastole (Fig. 2a and b). The time-averaged WSS at rupture point was only 2.7% of the value at the whole aneurysm dome (Table 1). The entire aneurysm dome showed low OSI except the area of rupture point. A markedly high OSI was found at the area of rupture point, which was seven times higher than that at aneurysm dome. (Table 1 and Fig. 2g). The intra-aneurysmal flow structure was relatively simple and stable comparing the blood streamlines between peak systolic and end diastolic (Fig. 2c and d). On the cutting plane, no concentrated flow impact on the area of rupture point at both peak systole and end diastole (Fig. 2e and f).

**Table 1:** Hemodynamic characteristics at rupture point and dome area

Hemodynamics	Rupture point	Dome
OSI	0.05686	0.00799
WSS at peak systole, pa	0.027	2.964
WSS at end diastole, pa	0.003	0.852
WSS time averaged, pa	0.046	1.722

*OSI* oscillatory shear index, *WSS* wall shear stress

## Discussion

In this study, we described a rare case that ruptured during angiography with confirmed rupture point and used CFD method to evaluate the hemodynamic characteristics at rupture point. Our results showed that the rupture point was associated with markedly low WSS and high OSI without flow impingement. Previous literature on hemodynamic characteristics of rupture point was reviewed (Table 2) and the results were controversial.

**Table 2:** Published articles of hemodynamic characteristics at rupture point

Reference	Case number	Rupture point identification	Hemodynamic results
Hodis et al. [3]	1	2D image	Concentrated impingement jet with elevated WSS and pressure at peak systole
Kono et al. [5]	1	Fused 3D image	Low WSS at end diastole and high pressure at peak systole
Omodaka et al. [9]	6	Inspection in surgery	Low WSS and high OSI
Fukazawa et al. [2]	12	Inspection in surgery	Low WSS and low flow-velocity pattern
Cebral et al. [1]	9	Inspection of extravascular blood in volume rendered 3D angiograms (*n* = 4) and/or additional CT images (*n* = 5)	The rupture site in 8 of 9 aneurysms (89%) had thinner and stiffer walls in regions of abnormally high WSS

*WSS* wall shear stress, *OSI* Oscillatory Shear Index, *2D* 2-dimensional, *3D* 3-dimensional, *CT* computed tomography

Hodis et al. [3] performed a similar study on an anterior communicating artery aneurysm, which also ruptured during the angiography. According to their results, a concentrated jet that impinged directly at the site of rupture, and elevated WSS and pressure were also found near the rupture site at peak systole. Another study by Kono et al. [4] also reported a rare case using CFD simulation. In that case, low WSS at end diastole and high pressure at peak systole were observed at the rupture site. As mentioned in that study, the possible mechanism of rupture is that low WSS at end diastole caused degeneration and thinning of the aneurysm wall and that high pressure at peak systole (impingement zone) resulted in rupture of the thinning wall. However, the rupture point in our present case did not shown direct flow impingement with low WSS through the cardiac circle. Omodaka et al. [5] and Fukazawa et al. [2] studied a total of 18 middle cerebral artery aneurysms with intraoperative confirmation of rupture point using CFD method. Similar results were obtained in both studies that the time-averaged WSS at the rupture point was significantly lower than that at the aneurysm wall without the rupture point. Recently, Cebral et al. [1] analyzed 9 aneurysms of which the rupture site could be identified in 3D images to investigate the effects of abnormal hemodynamics (either high or low WSS). According to their results, the models assuming wall thinning and stiffening in regions of abnormally high WSS were able to explain most of the observed rupture sites (8 of 9 cases).

The previous studies have demonstrated conflicting results about the local hemodynamic characteristics of the rupture site [1–5]. In our present study, a markedly low WSS and high OSI was associated with rupture point without high flow impingement. Our previous study evaluated the hemodynamic characteristics of aneurysms just before rupture and low WSS was also revealed to be associated with rupture of the unruptured aneurysms [6]. Low WSS and high OSI are known to cause dysfunction of endothelial surface inducing nitrous oxide, increase endothelial permeability and consequently promote inflammatory cell infiltration [7, 8]. These remodeling process might thin and weaken of the vessel wall leading to further rupture [9, 10]. Our findings in this study may provide insight into the mechanism of aneurysm rupture. However, the result of the present case might be unique and whether high or low WSS is the critical in aneurysm rupture remains controversial.

The conflicting simulation results might be affected by many factors, including the clot at rupture site, vasospasm, aneurysm location and method used for identifying the rupture points. Further studies with larger sample size are needed to investigate the hemodynamic characteristics of rupture point. Other limitations were also needed to be addressed. The hemodynamics in aneurysm before and after the rupture may be different for the consequence of a change in morphology [11]. The rupture from an unruptured aneurysm might be different from the mechanism of the rebleeding of a ruptured aneurysm. The hemodynamics is one of the important factors of aneurysm rupture and may not comprehensively explain the rupture mechanism. Other pathophysiological factors may also involve. The patient-specific inflow waveform was not available in this study, which might cause bias in simulation results. Some other assumptions, as rigid wall, laminar flow, and Newtonian blood might also induce bias.

## Conclusion

We present a rare case of which the rupture site was identified during angiography. The hemodynamic simulations revealed that the rupture point was associated with markedly low WSS and high OSI without flow impingement. The result may be unique to this particular aneurysm; however, our findings provide insight into the hemodynamics of rupture point.

## Abbreviations

CFD: Computational fluid dynamics
DSA: Digital subtraction angiography
LSA: Low shear area
OSI: Oscillatory shear index
WSS: Wall shear stress
